# Gross motor proficiency deficits among children and adolescents post posterior fossa brain tumor removal vs. traumatic brain injury in the chronic phase of recovery: a cross-sectional study

**DOI:** 10.3389/fspor.2024.1284421

**Published:** 2024-01-22

**Authors:** Sharon Barak, Amichai Brezner, Tamar Yissar, Etzyona Eisenstein, Shirley Ackerman-Laufer, Jana Landa

**Affiliations:** ^1^ Department of Nursing, Faculty of Health Science, Ariel University, Ariel, Israel; ^2^ Department of Pediatric Rehabilitation, The Edmond and Lily Safra Children’s Hospital, The Chaim Sheba Medical Center, Tel Hashomer, Ramat-Gan, Israel; ^3^ Sackler Faculty of Medicine, Tel Aviv University, Tel-Aviv, Israel

**Keywords:** BOT2, pediatric, brain injury, traumatic brain injury, motor performance, validity

## Abstract

**Introduction:**

Acquired brain injury (ABI) is a prevalent diagnosis in pediatric rehabilitation. Gross motor skills are often affected by ABI and limit the ability to participate in various physical activities. However, as ABI injury location is diverse, children and adolescents (youth) with localized ABI, such as ABI in the posterior fossa (ABI-PF) may present unique and different motor disabilities than youth with ABI on account of traumatic brain injury (TBI).

**Aims:**

The aims of the study were: (1) to compare gross motor deficits in youth with TBI vs. ABI-PF; and (2) to compare two methods on scoring BOT2 to determine which is better for identifying motor deficits.

**Methods:**

Participated in this study youth with TBI (*N* = 50) and ABI-PF (*N* = 30). Participants were tested on Bruininks-Oseretsky Test of Motor Proficiency-2nd Edition (BOT2) Upper-Limb Coordination, Balance, Strength, Running Speed and Agility, and Bilateral-Coordination subtests. Motor performance deficits were established using two-standard deviations (2SD) and age-equivalent methods. Between-group differences were assessed via independent *t*-tests and receiver operating characteristic curves (ROC).

**Results:**

According to the 2SD method, motor deficits in the ABI-PF group ranged from 20% to 66.66%, whereas in the TBI group 8%–16%. According to the age-equivalent method, in the TBI and ABI-PF groups 40%–66.0% and 46.66%–76.66% of the youth presented motor deficits, respectively. Moreover, ROC analysis showed that motor performance deficits of both groups in all sub-scales except for Bilateral Coordination differed enough to result in medium area under the curve.

**Conclusions:**

Motor deficits post-pediatric ABI are prevalent. In comparison to the TBI group, deficits are greater in the ABI-PF group. Moreover, compared to the 2SD method, the extent of motor deficiency is greater in the age-equivalent method. Therefore, using the later might provide a more valid classification of deficits in gross motor proficiency for youth post-ABI.

## Introduction

Acquired brain injury (ABI) is a prevalent diagnosis in pediatric rehabilitation (1) with an incidence of 661–1,035 per 100,000 (2). ABI can be classified into traumatic and non-traumatic (1). Traumatic brain injury (TBI) occurs when a sudden trauma results in damage to the brain (e.g., car accidents, falls) (3). Non-TBI cases include, for example, stroke, hypoxic/ischemic brain damage, infectious diseases or toxicity, and brain tumors. Brain tumors are the most common disease group of solid tumors in childhood (4). Moreover, tumors are the second most common form of cancer in childhood and almost half of all pediatric brain tumors arise in the posterior fossa (PF) (5).

In ABI, motor disabilities are often considered a less pervasive problem than psychosocial and cognitive deficits (6). Accordingly, it has been reported that 56%–72% of children and adolescents (youth) with ABI were able to ambulate independently at the time of discharge from the hospital (7). However, advanced gross motor skills (e.g., balance, static and dynamic postural control, speed and agility, coordination, and strength) which are important for high-level gross motor activities such as hopping, running and jumping are often affected by ABI. These problems remain long-lasting deficits that affect the higher motor performance of youth for years after ABI (8) and limit their ability to participate in various physical activities (3). However, as the ABI injury location is diverse, youth with localized ABI, such as ABI in the PF (ABI-PF) may present unique and different motor disabilities than youth with TBI. More specifically, damage because of PF tumors is usually localized to the cerebellar area, and the cerebellum is connected to many cerebral areas and its input regulates the excitability of cerebral motor control areas. Therefore, lesions to the cerebellum will affect the execution of various movements, specifically rapid, timed, and spatial dependent (9). Damage to the cerebellum may also result in balance and associative motor learning difficulties (10). In contrast, in TBI, this type of specific cerebellar damage is much less common, and the pathology commonly involves white matter tract damage because of diffuse axonal injury (11) as well as localized damage to specific cortical areas, most commonly to the frontal lobes. Accordingly, in comparison to ABI-PF, in TBI, it is a common finding that youth will have a higher level of motor function. Differences in motor function between the two groups may be enhanced by high-grade malignant tumor-related chemotherapy in youth with tumor-related ABI (12).

Despite the increased survival of youth with brain tumors, studies investigating objective motor functioning in this population are scarce (13). For example, Piscione et al. (14) study revealed significant differences between youth with brain tumors and normative population data for body coordination and strength and agility. Varedi et al. (15) observed balance impairments in 48% of adult survivors of pediatric central nervous system tumors. Specifically, within the pediatric PF tumor survivors, Piscione et al. (14) reported vermis infiltration of the tumor as a risk factor for lower body coordination scores and chemo and radiotherapy for lower strength and agility scores. In a more recent study by Decock et al., among youth with PF tumors (*N* = 56), motor performance deficits at the beginning of rehabilitation ranged from 5.35% (upper limb mobility) to 26.78% (balance). At the end of the rehabilitation, the prevalence of motor performance deficits ranged from zero percent (upper limb mobility) to 8.92% (balance) (16).

Several outcome measures are used to assess advanced motor skills performance in youth post-ABI (1). One of the most used outcome measures is the Bruininks-Oseretsky Test of Motor Proficiency 2 (BOT2) (17). The BOT2 is an evaluative eight-subtest standardized measure tool that assesses gross and fine motor proficiency (i.e., Fine Motor Precision, Fine Motor Integration, Manual Dexterity, Bilateral Coordination, Balance, Running Speed and Agility, Upper-Limb Coordination, and Strength) in youth aged four to 21 years old. The test was acknowledged as a supplementary measure to the core outcome measures for the evaluation of youth who sustained ABI (18). BOT2 can be used in two different ways to detect motor deficits. The first method utilizes the various subtests' individual point scores. A cutoff point of two standard deviations below the mean is commonly used to establish the presence of motor performance deficits (19). The second method utilizes the different subtests' age equivalents calculations. According to this method, youth can be grouped according to the gap between their chronological age and their motor performance age equivalent. The two different evaluation methods may provide different results concerning the motor proficiency level of youth with ABI. However, to the best of our knowledge, no previous study compared motor proficiency tests, including BOT2, different scoring systems effect on the prevalence of motor proficiency problems. There is however much debate in the topic regrading which type of scoring system is more appropriate in other populations and assessment domains. For example, when assessing writing skills, it has been suggested that the reliability for age-equivalent scores is much poorer for advanced test-takers (20). Accordingly, many assessments do not report age or grade equivalents beyond a specified age or grade level. For example, in the Oral and Language Written Scales (21), age-equivalent scores are not reported after age 12 and grade-equivalent scores are reported only up to grade six. The acquisition of writing skills occurs most rapidly during the early years because writing mechanics are taught in the primary grades. The degree of discrimination among examinees with advanced writing skills is demonstrated by smaller changes in score points (22).

Considering the knowledge on different scoring systems in other domains, a better understanding of the motor proficiency of youth with ABI and the impact of different scoring systems on it is important. Clinicians and researchers can use the BOT2 to screen for motor impairment, determine the need for further assessment/intervention, develop and evaluate motor training programs and make placement decisions regarding physical education programs (3). This study was therefore undertaken to: (1) compare gross motor deficits in youth with TBI vs. ABI-PF, and (2) compare two methods on scoring BOT2 to determine which is better for identifying motor deficits.

## Materials and methods

### Participants

Included in this cross-sectional study were: (1) youth (males and females) diagnosed with moderate-to-severe ABI. Diagnosis of ABI was conducted by medical doctors. Different tests and measures were conducted to diagnose and map ABI condition. Among these tests were image tests, namely, computerized tomography and/or magnetic resonance imaging. In addition, thorough medical history was obtained from the patient and/or family members. Specifically, for TBI, injury severity level was ranked using the Glasgow Comma Scale upon admission to the emergency room. Included in the study only youth with Glasgow Comma Scale of 3–12); (2) age range: 5–18 years old; (3) youth in the chronic phase of recovery (i.e., at least six months post-injury); and (4) youth who are able to follow simple three-step directions and commands (specifically: raising the arms, getting up from a chair and stopping an activity).

Conversely, participants were excluded from the study if they met any of the subsequent criteria: (1) inability to independently traverse a distance of at least 10 meters, either with or without orthotic assistance, (2) presence of pre-existing conditions that could impede motor performance, directly or indirectly related to the trauma, and (3) sustaining fractures that hindered the appropriate administration of theBOT-2.

All methodologies and procedures executed in the course of this study adhered strictly to pertinent guidelines and regulations. The study itself obtained ethical approval, including a waiver from the obligation of securing informed consent, from the ethics committee of Chaim Sheba Medical Center (Approval Code: 6504-19-SMC).

### Measures

BOT2 is a reliable and valid instrument of motor-skills performance, used in individuals ages four through 21 (17). The complete battery of BOT2 consists of 53 items classified into eight subtests: Fine Motor Precision, Fine Motor Integration, Manual Dexterity, Upper Limb Coordination, Bilateral Coordination, Balance, Running Speed and Agility and Strength (17, 23). This study focused on gross motor function, therefore, only the following BOT2 subtests were examined: Upper Limb Coordination, Bilateral Coordination, Balance, Running Speed and Agility and Strength.

### Procedure

This study is a retrospective cross-sectional analysis focusing on youth who were hospitalized in a pediatric rehabilitation due to moderate-to-severe ABI. All individuals admitted to the department underwent a thorough assessment following the department's standardized protocol. Subsequently, all pertinent data were documented in the patients' medical records. Upon obtaining ethical approval from the institution to carry out this study, a detailed review of the medical records of youth diagnosed with moderate-to-severe ABI was conducted. This review aimed to identify those who met the specific inclusion criteria for the study. Individuals meeting the inclusion criteria had their relevant demographic, clinical, and BOT2 data extracted and analyzed.

The BOT2 is administered routinely in the pediatric department by the department's physical and occupational therapists as part of a motor performance battery to every youth having sustained an ABI. The current study focuses only on the gross motor domains which are all evaluated by the department's physical therapists. All physical therapists involved in BOT2 assessment (*N* = 3) had at least five years of seniority in pediatric rehabilitation and a minimum of two years of experience in administrating the BOT2. In addition, all of them partook in BOT2 administration training, and inter-rater agreement was examined. As the gross motor section of the BOT2 may require a lot of effort on behalf of the evaluated youth, the evaluation was commonly conducted during the first evaluation section of the day in a quiet room. The test was conducted according to the BOT2 manual using the long form. Testing was conducted in one session lasting approximately 60 min. Per the BOT2 protocol, item raw scores were calculated for each item (e.g., the number of correct responses or the duration of an activity sustained). Following this, the total point score is calculated for each subtest. Subsequently, using tables provided in the manual, each total point score is converted to a scale score (mean = 15; standard deviation = 5). Finally, age-equivalent scores were also calculated (17).

### Data analysis

To evaluate for demographic and clinical characteristics bias, differences between the TBI and the ABI-PF groups in age, time post-injury, and gender were evaluated using independent *t*-tests and chi-square tests.

#### Gross motor performance deficits in youth with TBI and ABI-PF

The presence of motor performance deficits was established using the 2SD method. Following is a description of the 2SD method calculation. First, each item of BOT2 received a raw score. The raw scores' units in the various sub-domains assessed are as followed: Upper Limb Coordination—number of catches, throws and dribbles; Bilateral Coordination—number of jumps, touches, pivoting thumbs and feet and fingers taps; Balance—number of seconds or number of steps; Running speed and agility—number of seconds; and Strength—number of repetitions in a specified time unit and distance. Following, each raw score was converted into point score which ranges from 0 to 10, depending on the sub-scale. Finally, the point scores are converted to scaled scores which are based on norms of age and sex. Scaled scores mean score is 15 and the standard deviation is 5. A cutoff point of two standard deviations below the mean was used to establish the presence of apparent motor performance deficits (19). Accordingly, participants were grouped into those performing within and less than two standard deviations. This information was added to the data analysis section (19). In addition, age equivalents were obtained from the manual for each subject's performance on each subtest evaluated. For this study, three groups of performance deficits below chronological age were obtained: (1) up to 24 months (24), (2) 25–35 months, and (3) ≥36 months. First, the prevalence of motor performance deficits in the two assessment methods in the entire group (TBI + ABI-PF) was calculated. Next, deficits prevalence in each etiology group was calculated separately and compared using a chi-square test.

To better understand BOT2 ability to identify youth with motor proficiency on account of TBI vs. ABI-PF issues, receiver operating characteristics (ROC) curves were constructed. In ROC curves, the rate of true positive sensitivity was plotted on the y-axes, and the rate of false positives (1-specificity) was plotted on the x-axes (25). The area under the ROC curve (AUC) indicates the power of the instrument as it denotes the probability of the assessment to rank the child into the correct group. AUCs of 0.5–0.7, 0.7–0.9, and 0.9–1.0, represent low, medium, and high accuracy, respectively (26).

All data analyses were done using Statistical Package for Social Science (SPSS), version 29. ROC analysis was conducted using MedCalc statistical software, version 14.10.2.

#### *Post-hoc* power analysis

*Post-hoc* power analysis for the main (first) research question, pertaining to the differences between youth with TBI and ABI-PF in gross motor ability was conducted using G*Power (version 3.1.9.4). More specifically, the test evaluated the power of the study to detect significant between-group differences in motor ability deficits using each of the evaluation methods (age equivalent and two-standard deviation method). For this purpose, the test family used was “exact” with proportion inequality statistical test for two independent groups (Fisher's exact test), given an alpha, n, and effect sizes.

## Results

A total of *N* = 80 youth with ABI participated in the study (mean age = 11.34 ± 3.55 years; age range: 5.7–17.2 years); age range: 5.6–19; 60% boys). Most participants had TBI (*n* = 50). The mean Glasgow Comma Scale upon admission to the emergency room of youth with TBI was six and ranged from three-to-nine (moderate-to-severe injury). For information regarding group differences and additional information, refer to Table 1.

**Table 1:** Study participants’’ demographic and clinical characteristics.

Variables			Entire group (*n* = 80)	ABI-PF group (*n* = 30)	TBI group (*n* = 50)	Between groups differences: Chi square test OR *t* statistic (*p*-value)
Age, years: mean (SD)			11.34 (3.55)	10.17 (3.33)	11.78 (3.56)	1.708 (0.009)
Sex	Girls: *n* (%)		28 (34.14)	8 (26.66)	15 (30.00)	0.003 (0.95)
Time post injury, years: mean (SD)			3.03 (2.71)	3.33 (2.51)	2.91 (2.78)	0.653 (0.49)
Injury cause-	TBI	Car accident, *n* Fall, *n* Other, *n*	–	–	35 (70.00) 5 (10.00) 10 (20.00)	–

ABI-PF, acquired brain injury-posterior fossa; SD, standard deviation; TBI, traumatic brain injury.

### Gross motor performance deficits in youth with TBI in comparison to ABI-PF

The prevalence of motor performance deficits in the two standard deviation method in the entire group (TBI + ABI-PF) ranged from 16.25% (Bilateral coordination) to 35.00% (Balance). Motor performance deficits were greater when using the age equivalent method and ranged from 75.00% (Balance) to 92.50% (Bilateral coordination). Using the age-equivalent method the prevalence of total motor performance deficits was statistically significantly greater in the ABI-PF group vs. the TBI group only in the Balance sub-scale (Chi-squared = 8.2 (*p* = 0.04). When using the two-standard deviation method the prevalence of deficits was greater in the ABI-PF vs. the TBI group in the following sub-domains: Upper Limb Coordination (Chi-squared = 24.7, *p* < 0.01), Balance (Chi-squared = 20.4, *p* < 0.01), Running Speed and Agility (Chi-squared = 6.9 (*p* = 0.008), and Strength (Chi-squared = 13.6 (*p* < 0.01). For additional information, refer to Table 2.

**Table 2:** Prevalence of motor performance deficits according to the age equivalent method.

		Total group (*N* = 80)	Acquired brain injury—posterior fossa (*N* = 30)				Traumatic brain injury (*N* = 50)				Between-group differences in total deficits
		Total deficits: *n* (%)	≤24 months of chronological age (%): *n* (%)	24–36 months below chronological age (%): *n* (%)	≥36 months below chronological age (%): *n* (%)	Total deficits: *n* (%)	≤24 months of chronological age (%): *n* (%)	24–36 months below chronological age (%): *n* (%)	≥36 months below chronological age (%): *n* (%)	Total deficits: *n* (%)	Chi-squared (*p*-value)
Upper-Limb Coordi-nation	Age equivalent	70 (87.50)	3 (10.00)	8 (26.66)	16 (53.33)	27 (90.00)	14 (28.0)	9 (18.0)	20 (40.0)	43 (86.00)	0.2 (0.60)
	Two standard deviations method	20 (25.00)	–	–	–	14 (46.66)	–	–	–	6 (12.00)	**24.7** (**<0.01)**
Bilateral Coordi-nation	Age equivalent	74 (92.50)	13 (43.33)	0	14 (46.66)	27 (90.00)	10 (20.0)	4 (8.0)	33 (66.0)	47 (94.00)	0.4 (0.51)
	Two standard deviations method	13 (16.25)	–	–	–	6 (20.00)	–	–	–	7 (14.00)	0.4 (0.48)
Balance	Age equivalent	60 (75.00)	0	5 (16.66)	23 (76.66)	28 (93.32)	4 (8.0)	6 (12.0)	31 (62.0)	32 (64.00)	**8.2** (**0.04)**
	Two standard deviations method	28 (35.00)	–	–	–	20 (66.66)	–	–	–	8 (16.00)	**20.4** (**<0.01)**
Running Speed and Agility	Age equivalent	72 (90.00)	3 (10.00)	5 (16.66)	20 (66.66)	28 (93.32)	9 (18.0)	4 (8.0)	31 (62.0)	44 (88.00)	0.50 (0.47)
	Two standard deviations method	21 (26.25)	–	–	–	13 (43.33)	–	–	–	8 (16.00)	**6.9** (**0.008)**
Strength	Age equivalent	69 (86.25)	3 (10.00)	3 (10.00)	21 (70.00)	27 (90.00)	12 (24.0)	8 (16.0)	22 (44.0)	42 (84.00)	0.5 (0.45)
	Two standard deviations method	17 (21.25)	–	–	–	13 (43.33)	–	–	–	4 (8.00)	**13.6** (**<0.01)**

Bold values signify statsitcialy significant between-group differences in total deficits (*p* < 0.05).

When looking separately at the TBI group, according to the two-standard deviation method, motor deficits ranged from 8.0% (Strength) to 16.0% (Balance and Running Speed and Agility). Motor deficit prevalence in the ABI-PF was higher and ranged from 20.0% (Bilateral Coordination) to 66.66% (Balance). In contrast, the age-equivalent method revealed higher rates of motor deficits. In the TBI group, 40% (Upper Limb Coordination) to 66.0 (Bilateral Coordination) of the youth presented motor function that is ≥36 months below their chronological age. In the ABI-PF group, 46.66% (Bilateral Coordination) to 76.66% (Balance) of the youth presented such motor deficits (see Table 2).

**Figure 1:**
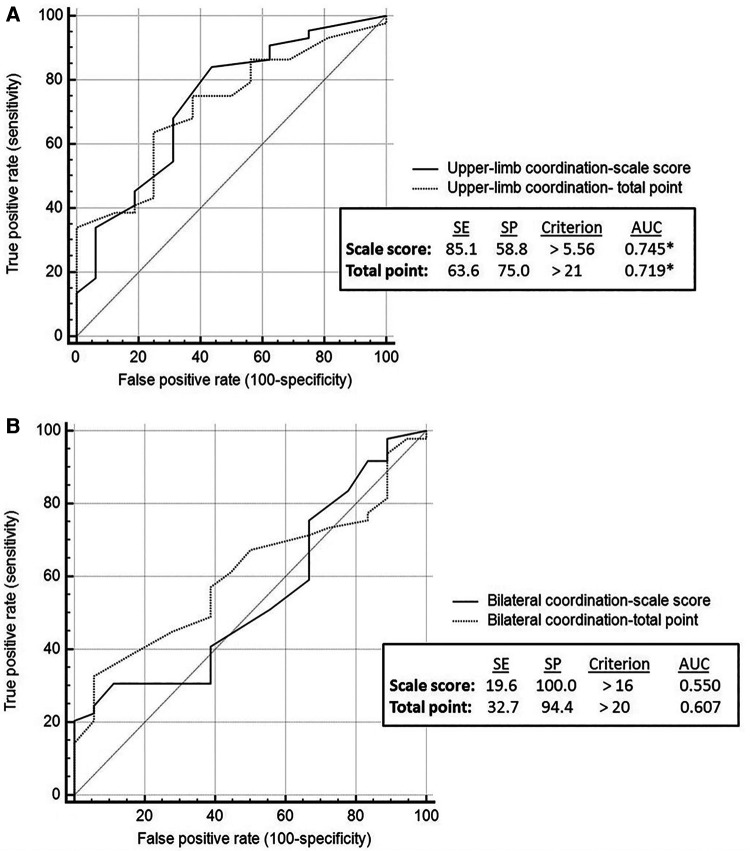
Coordination sub-scales: discriminative ability by brain injury etiology. Discriminative validity of upper limb coordination sub-scale (**A**). Discriminative validity of bilateral coordination sub-scale (**B**). AUC, area under the curve; SE, sensitivity; SP, specificity.

**Figure 2:**
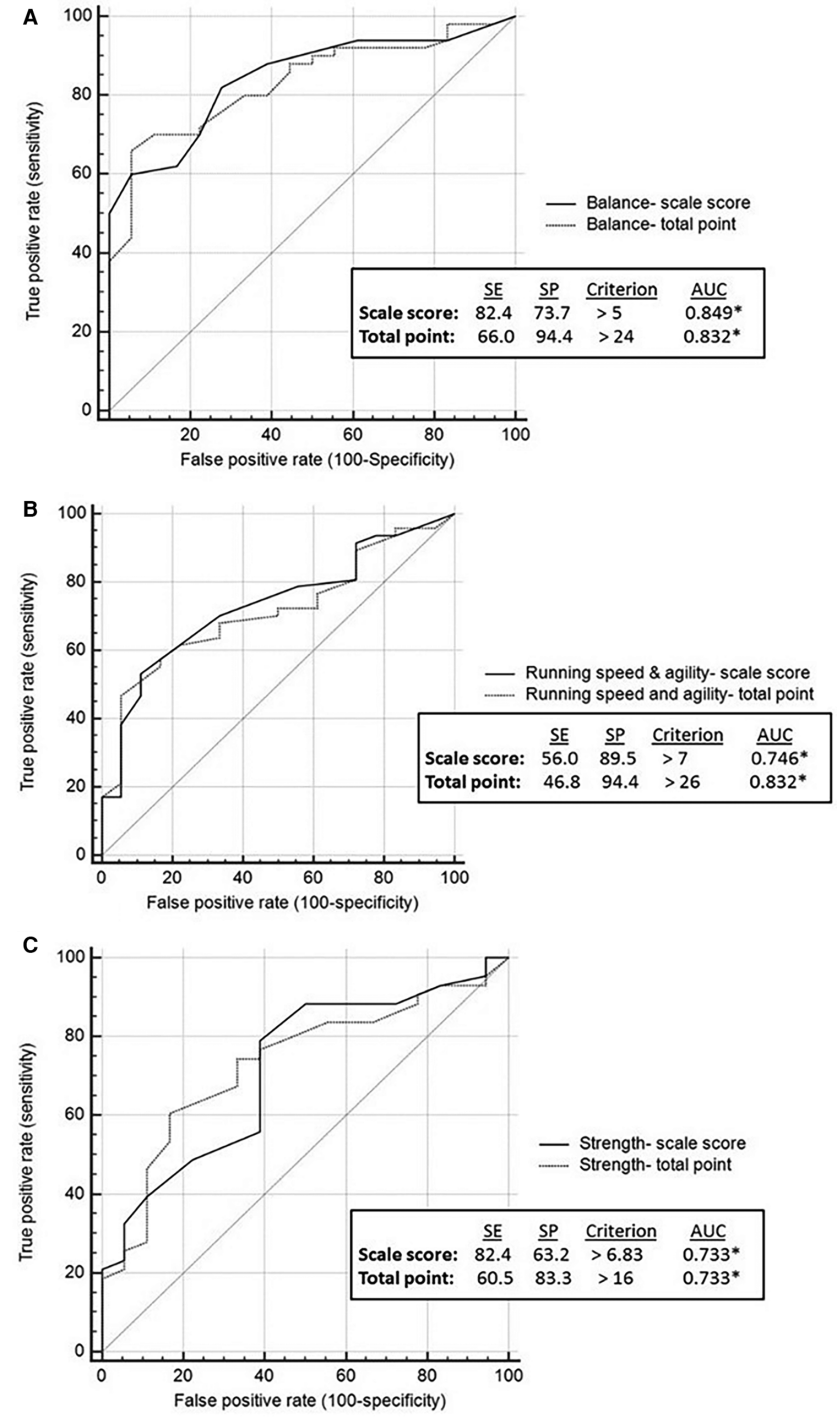
Balance, running speed and agility and strength: discriminative ability by brain injury etiology. Discriminative validity of balance sub-scale (**A**). Discriminative validity of running speed and agility sub-scale (**B**). Discriminative validity of strength sub-scale (**C**). AUC, area under the curve; SE, sensitivity; SP, specificity.

ROC analysis showed that all BOT2 sub-scales, except for Bilateral Coordination, demonstrated medium accuracy in differentiating between youth with PF and non-PF injuries (AUC = 0.7–0.9). Bilateral Coordination accuracy is considered low (AUC < 0.7) (Figures 1A–B, 2A–C). Meaning, motor performance deficits of both groups in all sub-scales except for Bilateral Coordination differed enough to result in medium AUC.

### *Post-hoc* power analysis

*Post-hoc* power analysis for this section revealed that when using the age equivalent method, in which both study groups presented high prevalence of motor performance deficits, the mean power of the study was not sufficient (power = 0.40). However, for the two-standard deviation method, the power was good and equals to 0.85.

## Discussion

The main aims of the study were to increase knowledge on gross motor performance deficits in youth with ABI and specifically in youth post TBI vs. ABI-PF and examine the influence of motor assessment methods on deficits prevalence. In addition, BOT2's ability to discriminate between youth with different ABI etiologies was examined. Understanding motor performance post-pediatric ABI is of special importance as motor deficits may result in difficulties in making and keeping personal relationships, in difficulties in taking part in social activities and in limitations to participating in recreational or leisure activities. In the following section, we discuss the results of each of the aims.

### Gross motor performance deficits after ABI

The data showed that deficits in motor performance are present in the chronic phase in youth with moderate-to-severe ABI. According to the two-standard deviation method, the prevalence of deficits in the different BOT2 sub-domains ranged between 20.0% and 66.66% in the ABI-PF group and 8.0%–16.0% in the TBI group. The prevalence of motor deficits in both study groups were considerably higher in the age equivalent method (>90% and >64% of youth in the ABI-PF and TBI groups, respectively). The differences between the two methods regarding the prevalence of motor deficits post-ABI is important as according to Deitz et al. (23) the two-standard deviations method might be one of the criteria for receiving therapy services in certain programs. Therefore, using the two-standard deviations method to identify motor prevalence would not be accurate enough as a child with a score that falls between 1 and 2 standard deviations below the mean, will not be qualified for therapeutic services while he/she might still present significant motor deficits in comparison to his/her peers—preventing him/her from participating in common or mutual activities. Moreover, as among the TBI group, most children score within two standard deviations from the norm's mean in all domains assessed (Table 2). Therefore, it may be appropriate to use a more challenging motor assessment in this sub-group of children with ABI. For instance, Wong and colleagues (27) developed a 20 items scale (the Acquired Brain Injury Challenge Assessment) to assess the advanced motor skills of children with ABI. The authors reported that the new assessment tool displayed excellent reliability and initial evidence of validity. Another assessment of interest in children and adolescents with TBI is the High-level Mobility Assessment Tool (HiMAT). The HiMAT demonstrated excellent inter-rater reliability, re-test reliability and responsiveness to change among children and adolescents with TBI (*N* = 52) (28).

The age-equivalent method also possesses several limitations. For example, the use of age curve fails to consider the variation in the curve. Consequently, a child may achieve an age-equivalent score at a level lower or higher than their chronological age, however, this level of performance may be within the normal bounds of performance for a child of that age (29). Despite the disadvantages associated with the age-equivalent method, age-equivalent scores can still be more useful because they provide an estimate of the child's absolute level of performance (30).

### Sub-domains with most significant deficits

Balance—The sub-domain with the highest prevalence of deficits in both study groups is balance. More specifically, in the ABI-PF group 66.66% (two standard deviations method) to 93.32% (age equivalent method) of the group had deficits in balance. In the TBI group, the prevalence ranged from 16% to 64% (two standard deviation and age equivalent method, respectively). Other authors also reported balance deficits post-ABI (24). Long-lasting balance problems post-ABI is not surprising. In the ABI-PF group, damage to the cerebellum can cause balance deficits. More specifically, evidence indicates that certain areas of cerebellar cortex and nuclei (e.g., cerebellar vermis and fastigial nucleus) appear to be engaged in numerous functions, including, balance/vestibular behaviors (31, 32). In the TBI group, balance problems may be explained by white matter lesions. More specifically, in a study of *N* = 507 youth with TBI, widespread disruption in white matter organization was observed following complicated mild to severe TBI. These alterations appear to persist and encompass a larger number of white matter regions with time post-injury. The corpus callosum appears to be particularly vulnerable to injury, an effect that persists years post-TBI (33). Such white matter changes may disrupt important cortical-subcortical connections that assist in motor control and balance (34).

Slowness—slowness as evaluated in the Running Speed and Agility is another domain in which youth with ABI-PF and TBI exhibited significant difficulties. Accordingly, cognitive response speed deficits and cognitive motor speeded performance deficits (i.e., such as speeded hand function tests and finger tapping) (19) had previously been reported as a problem and a prominent characteristic post-ABI (35–37). Slowness also commonly manifests in gait. For instance, Schaaf et al. (1997) (7) reported that in repeated gait analysis, ambulatory youth with ABI demonstrate significant reductions in velocity and cadence and in other motor activities which require speed. Slowness in the TBI group can be explained by the diffuse axonal injury, damage to the frontal cortex (e.g., premotor cortex, supplementary motor area), and basal ganglia. In the ABI-PF group, slowness may be caused by damage to cerebellar controlling rhythmic movements areas (38). As with the deficits seen in balance, slowness may hinder youth's post-ABI ability to reengage in school and community activity (3), specifically, the ability to engage in physical activities in the same pace with their peers.

### Interventions and physical activity for improving motor ability

Considering the impact of motor performance deficits in real life, it is also important to explore the extent to which interventions improve motor function. For example, based on a systematic review of the impact of physical therapy intervention on balance post-TBI (*N* = 259), the evidence about the effects of the physical therapy interventions in improving the balance ability was limited (39). In another systematic review on the effectiveness of interventions on gross motor outcomes of youth with an ABI, the authors concluded that although the included studies demonstrated preliminary evidence for a positive effect on gross motor outcomes following the interventions, low study methodological quality indicates that care is needed when interpreting and generalizing results to youth with an ABI (40, 41)

Regarding physical activity, the results of this study show that both groups of youth, and especially the ABI-PF group, have low level of motor ability. Hence it is important to adapt activities in therapy and community-based activities to enable the child to partake in it (42). There are four main aspects that can be changed to adapt the activity: (1) Teaching or coaching style—pertains to how the instructor delivers the activity. For example, using appropriate physical assistance; (2) Rules changing (e.g., allowing for more bounces of ball); (3) Changing the environment (e.g., making changes to the activity space); and (4) Allow the use of equipment (e.g., changing the devices used to play the game) (42–44).

Our study has several limitations to generalization. First, study participants consisted of youth during the chronic phase of ABI recovery (≥6 months post-injury). Therefore, our findings may not be generalized to individuals during the acute and sub-acute phases of recovery. Second, in the current study, we have a relatively heterogenic group in terms of age. Therefore, for better generalizability, future studies with larger sample sizes are warranted. Finally, future studies should aim at exploring factors associated motor performance deficits in the chronic phase of TBI and ABI-PF recovery.

## Conclusion

Motor deficits years post-ABI in youth are prevalent. However, in comparison to the TBI group, deficits are more severe and more prevalent in the ABI-PF group. Moreover, in comparison to the two-standard deviations method, the extent of motor deficiency is greater when using the age-equivalent method in both study groups. Therefore, using age-equivalent method might provide a more valid classification of deficits in gross motor proficiency for youth post-ABI. The results also suggest that motor performance deficits of both groups in all sub-scales except for Bilateral Coordination differed enough to result in medium AUC. Consequently, BOT2 is a valid measure of physical performance for youth in the chronic phase of ABI. It is important to consider the child's post-ABI motor deficits as they might have cardinal implications on their ability to reenter school and partake in activities with their peers, beyond their deficits in cognitive and academic functions. Study's results may contribute to clinicians and therapeutic sports experts, as knowledge on motor abilities of youth post-ABI is important when choosing intervention/activity type.

## Data Availability

The data analyzed in this study is subject to the following licenses/restrictions: Data is available upon request. Requests to access these datasets should be directed to Sharon Barak: sharoni.baraki@gmail.com.
